# Innovation practices for survival of small and medium enterprises (SMEs) in the COVID-19 times: the role of external support

**DOI:** 10.1186/s13731-021-00156-6

**Published:** 2021-05-27

**Authors:** Nawal Abdalla Adam, Ghadah Alarifi

**Affiliations:** College of Business and Administration, Princess Nourah bint Abdulrahman University, Riyadh, Saudi Arabia

**Keywords:** Small- and medium-sized enterprises (SMEs), SME performance, SME survival, Coronavirus (COVID-19) crisis, Innovative practices, External support

## Abstract

Global epidemic crises, such as the coronavirus (COVID-19), usually expose small and medium enterprises (SMEs) to various kinds of challenges and may put their lives at risk. This study aims to develop a theoretical model to provide insights about the association between innovation practices and the SMEs’ performance and survival while underlining the auxiliary role of external support in such a relationship. Online questionnaire has been used to collect the data from 259 randomly selected SME managers in Saudi Arabia, and the data was analyzed using the SmartPLS3 software. The structural equation modeling results showed that the innovation practices adopted by SMEs to face the repercussions of COVID-19 had a positive impact on the performance and likelihood of business survival. PLS-SEM bootstrap results indicated that external support aids strengthen the positive impact of SMEs’ innovation practices on business survival rather than its performance. The study has several significant practical implications for SME managers, governments, and policy makers that have been stated.

## Introduction

Since its emergence in late 2019, the COVID-19 epidemic has caused negative effects on the economies of countries and has had a disastrous impact on human health. The conditions and restrictions imposed in most countries to limit the virus’s spread among people, such as social distancing and quarantines, have led to distortions in the system of supply and demand for goods and slowed many countries’ economies. The repercussions of the COVID-19 pandemic have been felt across all economic sectors and institutions, including small and medium enterprises (SMEs) (Hasanat et al., 2020).

Studies have shown that SMEs are confronted with various difficulties and challenges due to the COVID-19 pandemic. The period of closure and movement prevention policies adopted by governments in many countries have greatly affected SMEs, paralyzing their operations, weakening their financial positions, and exposing them to financial risk (Omar et al., 2020; Oyewale et al., 2020). SMEs have suffered from a shortage of workers and production inputs because of distortions that marred supply chains, which negatively affected their sales (Gurría, 2020; Segal & Gerstel, 2020) and their ability to fulfill their financial obligations and pay employees’ salaries (Robinson & Kengatharan, 2020). This problem has coincided with a decrease in consumer spending because of the reduction in consumers’ income and widespread feelings of uncertainty (Gurría, 2020). As a result, many SMEs found themselves incapable of dealing with the situation (Ozili, 2020). Some businesses have stopped their activities and remained closed since the first months of the outbreak (Bartik et al., 2020).

Published research has indicated that SMEs have failed to withstand the consequences of economic crises (Latham, 2009; Michael & Robbins, 1998). This defect can be attributed to a lack of financial resources and the high cost of business capital (Domac & Ferri, 1999) as well as limited administrative and technical capacities (Demirgüç-Kunt et al., 2005). Researchers have emphasized that SMEs are often the enterprises most affected by economic crises (Latham, 2009; Robbins & Pearce II, 1993). Therefore, a socioeconomic crisis related to people’s health such as the COVID-19 pandemic can be expected to have dire effects on SMEs because these businesses require strong connections with people, whether they are customers or suppliers (Nugent & Yhee, 2002).

To protect this vital sector from collapse due to the COVID-19 crisis, many governmental and nongovernmental organizations (NGOs) have provided various forms of support to SMEs. Governments have adopted several policies that will mitigate the negative effects of this crisis (Ahmad et al., 2020). SMEs have received some financial support from local and international NGOs and financial institutions during the COVID-19 crisis (Song et al., 2020). Additionally, SME owners have adopted a number of practices and strategies to confront the ramifications of the crisis (Thorgren & Williams, 2020). During the early outbreak of the pandemic, authors expected that SMEs’ responses and practices would focus on financial spending reductions (Thorgren & Williams, 2020), digital technology exploitation (Guo et al., 2020; Indriastuti & Fuad, 2020; Papadopoulos et al., 2020), and disaster management (Eggers, 2020).

Previous studies on SMEs’ practices in response to the COVID-19 pandemic and business performance have examined the impact of each practice on business performance separately (Gerald et al., 2020; Guo et al., 2020; Indriastuti & Fuad, 2020; Sobaih et al., 2021). For example, Guo et al. (2020) found that the use of digital technology helps SMEs to survive and cope with the consequences of the pandemic. Their findings call attention to the importance of information technology in helping SMEs cope with the challenges created by the COVID-19 crisis. Similarly, Gerald et al. (2020) argued that practicing strategic agility mitigates the negative effects of the COVID-19 crisis on SMEs’ performance. These findings take a managerial approach to SMEs’ practices for responding to the crisis.

However, a limited number of studies have focused on SMEs’ practices for survival after the outbreak of the COVID-19 pandemic (Omar et al., 2020). Omar et al. (2020) showed that SMEs have used financial and marketing strategies for survival when faced with the repercussions of the COVID-19 crisis. Their findings are significant because they focused on SMEs’ long-term, rather than short-term, performance. However, the impact of these strategic responses on SMEs’ long-term performance and their potential for efficiency needs further study.

Moreover, studies have been deficient in addressing the impact of external support received by SMEs since the COVID-19 outbreak on their performance and survival (with the exception of Song et al., 2020). As such, this study examined the effectiveness of SMEs’ innovation practices in response to the challenges posed by the COVID-19 pandemic. More specifically, the present research focused on the impact of SMEs’ innovation practices on business performance and survival. The current research also examined the moderating effect of external support in the relationship between SMEs’ innovation practices and business performance and survival. Focusing on the marketing and organizational innovation practices adopted by SMEs in Saudi Arabia to face the threats created by the COVID-19 pandemic, the present study is based mainly on the hypothesis that SMEs’ innovation practices in times of crisis, such as the COVID-19 pandemic, may help increase the organization’s performance and, subsequently, ensure its survival.

This study contributes to the growing literature on SMEs’ practices and external support in times of crisis and provides additional insights for SME managers and policymakers about the importance of external support in strengthening the positive impact of innovation practices on business survival.

After reviewing the literature on SMEs’ innovation practices, performance, survival, and external support, we will introduce our theoretical framework and analysis of the five hypotheses being tested. We then present and discuss the results of our data analysis to highlight our main conclusions.

## Literature review

### Innovation practices and SME performance

Innovation has become a necessity for all contemporary enterprises that want to survive in a world characterized by competition, technological change, and recurring crises. The concept of innovation refers to the use of new technology or new management practices in an organization to achieve a targeted improvement in its operations (Tornatzky et al., 1990). From a SME perspective, innovation commonly indicates new products or processes that address customer needs more competitively and profitably than existing ones (O’Regan & Ghobadian, 2006; Zahra et al., 1999). We use the term “innovative practices” in this study to refer to the effective implementation of new solutions to challenges faced by SMEs, which include effective implementation of new ideas in relation to the organization’s product, services, or processes; new marketing mechanisms; or new administrative practices for work amelioration and upgraded performance (Damanpour, 1992; Johannessen et al., 2001; OECD/Eurostat, 2005).

The key driver of innovation practices in enterprises is the ambition to get reimbursement in the form of better performance. Therefore, innovation is defined as creation of some modifications in the enterprise’s practices that are intended to obtain an improvement in performance (Curristine, 2006). Based on the literature, performance in this study is defined as achieving the institution’s objectives related to sales, profitability, competition, market share, and any other strategic goals (Hult et al., 2004). Researchers also defined performance as achieving a set of desired outcomes resulting from the realization of the marketing objectives (Chittithaworn et al., 2011). For Yıldız et al. (2014), performance refers to an effectiveness in carrying out the enterprise’s tasks, which results in achieving its stated objectives. Achieving high performance level implicitly indicates enterprise success (Mahmudova & Kovács, 2018). Measuring the enterprise’s performance helps to enhance the positive aspects of its operation and provides an opportunity to take corrective measures to address weaknesses (Mahmudova & Kovács, 2018).

There is a large amount of literature supporting the significant positive relationship between innovation and SME performance (Qian & Li, 2003; Rosenbusch et al., 2011; Verhees & Meulenberg, 2004; Yıldız et al., 2014). The published research also indicated the positive impact of innovation capabilities on SME performance (O’Cass & Sok, 2014; Oura et al., 2016; Zhang, et al., 2018). Zulu-Chisanga et al. (2016) noted that the efforts exerted to develop different innovations are the primary reason for the improvement in SMEs’ financial indicators. Previous studies also indicated the positive correlation between the innovation capabilities and SMEs’ performance (O’Cass & Sok, 2014; Oura et al., 2016; Zhang et al., 2018). Freeman (2004) added that distinct SMEs’ performance is an outcome of the effective implementation of innovations. However, Lin and Chen (2007) argued that the impact of managers’ innovation practices on SME income outweighs that of technological innovation. Therefore, we argue that the innovation practices of SMEs in all environmental situations such as the COVID-19 pandemic can contribute positively to enterprise performance. Therefore, we hypothesize the following:

H1: SME’s innovation practices have a significant positive impact on its performance.

### Innovation practices and SME survival

Enterprise survival was used in the current study to indicate the amount of time the enterprise takes to carry out its activities since its inception up to closure (Bercovitz & Mitchell, 2007). There are many parties in the community who benefit from the enterprise’s survival aside from its managers. They include workers, consumers, and suppliers (Bercovitz & Mitchell, 2007). Researchers confirm that enterprise survival is one feature of its performance (Danes et al., 2008; Kalleberg & Leicht, 1986). An enterprise can survive if it can adapt to the conditions and its surrounding environment (Child, 1972; Pfeffer & Salancik, 1978). Compared to large enterprises, SMEs have shorter life, more profitable, and largely affected by external environmental factors (Carroll & Huo, 1986). Some researchers consider survival to be an objective measure of enterprise success (Miner, 1997).

In times of crisis, the existences of SMEs are in danger (O’Reilly III & Tushman, 2011). Crises weaken SMEs’ growth and threaten their projects because their negative impact extends to all elements of the external enterprise environment (Dhochak & Sharma, 2015). For instance, in time of crisis, SMEs have limited financing opportunities due to weak capital market performance, lack of sufficient information, and component defects throughout the economy (Bester & Hellwig, 1989; Binks et al., 1992; Cowling et al., 2012; Hillier & Ibrahimo, 1993; Mason & Harrison, 2015).

The business innovations–survival relationship has been illustrated in numerous studies. Innovation is critical to the continuity of any enterprise (Ortiz-Villajos, 2014). According to Gaynor (2002), innovation is the core factor behind the survival and continuity of enterprises; it supports the company’s expansion and growth and enhances the enterprise’s future success. Previous studies suggested using innovations to overcome the obstacles and challenges of industrial SMEs’ success and survival (Bruns & Stalker, 1961; Hurley & Hult, 1998; Porter, 1990; Schumpeter & Redvers, 1934). Schumpeter (1942) declared that the enterprise’s survival is strongly linked to its innovation practices. Several studies have attempted to explain this link by pointing to some concepts relevant to both innovation and enterprise survival. For instance, a competitive advantage is simultaneously a product of enterprise innovation practices and a fundamental pillar of its survival (Brüderl et al., 1992; Cefis & Marsili, 2003; Helmers & Rogers, 2010). Schumpeter (1942) argued that enterprises cannot survive and continue their activities without being innovative. However, survival also results from achieving victory in the face of crises imposed by the external environment (Aldrich, 1979; Hannan & Freeman, 1977; Kanter & Brinkerhoff, 1981). Therefore, this study argues that the various innovation efforts exerted by SMEs for mitigating the negative effects of the COVID-19 pandemic can bring positive results to these enterprises. Therefore, the second hypothesis of this research is stated as such:

H2: SME’s innovation practices have a significant positive impact on its performance.

### SMEs and external support

External support refers to the assistance provided to the enterprise by external parties (Global, I, 2018). SMEs are increasingly using external support (Bennett & Robson, 1999, 2003) because it provides them with the essential knowledge and information necessary to strengthen their competitive position and increase their chances for future prosperity (Bennett & Robson, 1999; Penrose, 1959a, 1959b; Teece, 2002; Teece et al., 1997). Governments, advocates, and different agencies and institutions offer external support to SMEs to save their lives, boost their growth, stimulate innovation, and enhance their capabilities by increasing managerial capabilities and improving marketing skills, thereby ensuring they make a greater business contribution to the national economy (Chrisman & McMullan, 2004; Mason & Brown, 2013). Governments, on the other side, encourage SMEs to take external support to be better able to exploit their business capacity, improve their performance, increase their competitiveness, and assist in business expansion and growth (Cliff, 1998; Gimeno et al., 1997; Storey et al., 2010).

SMEs’ external support can be either direct or indirect. Direct external support usually takes the form of financial aid that is to be used in the acquisition of assets, the purchase of technology, or the implementation of development plans with the aim of solving funding deficiency problems. It is usually provided according to specific government policies or financial intermediary conditions (Freitas & Von Tunzelmann, 2008; Nishimura & Okamuro, 2011). Indirect external support usually takes the form of consultancies, ideas, and advice provided by experts, advisory offices, and educational institutions to help eliminate the lack of knowledge and increase the available amount of information (Freitas & Von Tunzelmann, 2008; Metcalfe & Ramlogan, 2005). Despite the diversity and the importance of external support for SMEs, researchers have noticed that SMEs benefit little from this support due to the lack of information and awareness about this form of support and the enterprise management’s inability to choose the appropriate type of support (Story, 1994).

External support is important for SMEs because it provides them with the knowledge needed to develop and implement innovations. According to Based  Woodman et al. (1993) innovations are usually grounded on the business informational support received from the enterprise surrounding environment. Cohen and Levinthal (1990) showed that innovations in enterprises result from combining the knowledge received from their external environment with their available internal knowledge. Damanpour (1991) confirmed a positive association between external support received by an enterprise and innovation. External support also provides an enterprise with human and financial resources that undergird innovation within the enterprise (Amabile, 1996; Scott & Bruce, 1994).

Numerous researchers have examined the linkage between external support and enterprise performance. For instance, Kent (1994) noted that the use of external support raises the SME’s financial indicators. Larsson et al. (2003) concluded that external support in the form of managerial consultancies and advice received by SMEs positively affect the business growth. Brush and Vanderwerf (1992) and Storey (2002) demonstrated that external consultants positively affected an SME’s performance, expansion, and viability. Research findings noted that the use of external support contributes positively to a business’s competitive advantage (Penrose, 1959a, 1959b; Teece et al., 1997). Dollinger (1985) emphasized the positive results of interaction with the external environment components on the SME’s performance. Many other scholars have emphasized the positive association between an enterprise’s performance and its use of external support (Bylund & McCaffrey, 2017; Matlay et al., 2005).

However, researchers asserted that the link between innovation practices and enterprise performance requiring an auxiliary factor represent a moderating variable (Covin & Slevin, 1989; Jones & de Zubielqui, 2017; Li & Atuahene-Gima, 2001). According to Rosenbusch et al. (2011), this moderator is expected to come from the enterprise’s external environment.

Studies have proven the strong and positive link between an enterprise’s performance and its likelihood of its survival (Friedlander & Pickle, 1968; Gibson et al., 1973; Steers, 1975; Thompson & McEwen, 1958). For example, Wiklund and Shepherd (2011) asserted that an enterprise survival requires a minimum level performance. In addition, published literature shows that an enterprise’s survival indicates its outstanding performance over a long period (Friedlander & Pickle, 1968; Gibson et al., 1973; Steers, 1975; Thompson & McEwen, 1958). Moreover, both an enterprise’s performance and survival are affected by external environmental factors and crises such as the COVID-19 pandemic (Holmes et al., 2010). This study argued that external support received by SMEs for the purpose of mitigating the effects of the COVID-19 pandemic contributes to strengthening the relationship between both innovation practices and an enterprise’s performance on the one hand and between innovation practices and the business survival on the other hand. Thus, we hypothesize the following:

H3: The positive association between an SME’s innovation practices and its performance may be stronger when the enterprise receives more external support.

H4: The positive association between an SME’s innovation practices and its survival may be stronger when the enterprise receives more external support.

## Theoretical framework

**Fig. 1:**
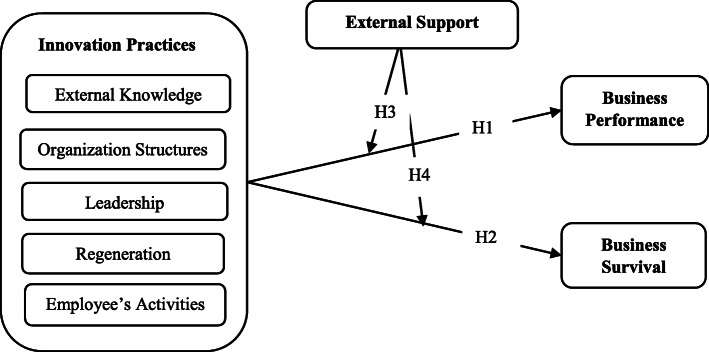
The conceptual research model

The conceptual framework in Fig. 1 shows the links between a SME’s innovation practices, the business’s performance, and survival. The model suggests that both relationships (innovation practices–performance and innovation practices–survival) are affected by external support. In addition, SME performance affected the business’s survival. Innovative practices (independent variable) shown in the model included six dimensions (external knowledge, structures, leadership, regenerations, employees’ activities, and marketing activities) extracted from the literature review. The dependent variables were the SME performance and SME survival. The model proposes that the connection between dependent variables and the independent variable is moderated by the external support.

First, the direct relationship between innovation practices and the SMEs’ performance is analyzed (H1). Then, the direct association between SME innovation practices and business survival is considered (H2). An analysis of the effect of external support on the strength of the relationship between SMEs’ innovation practices and their business performance is performed (H3). Finally, the role of external support in the link between innovation practice constitutions and SME survival is scrutinized (H4).

In the next section, the hypothetical relationships of the independent variable and dependent variables in the model are discussed.

## Methods

### Data collection and sample

The current study is limited to SMEs in Saudi Arabia that employ a number of employees, ranging between six and 250, with revenue less than 200 million Saudi Riyals. The online questionnaire prepared through SurveyMonkey was used to collect the data from SME managers. The online questionnaire is less expensive and helps obtain large responses in a short period of time (Bryman & Bell, 2014). The questionnaire was first translated from the English language into the Arabic language so the respondents could understand the questions. Then, an e-mail containing a link to the questionnaire was sent to a randomly selected sample frame, which contained a request to fill out the questionnaire and an explanation of its purpose. The participants were given 15 days to complete the questionnaire.

The online survey distributed during the last 2 weeks of August 2020 included 500 randomly selected SME managers (selected from the General Authority for Statistics [GaStat] database). At the end of the survey period, 259 participants completed the questionnaire, resulting in a response rate of 52%. The total number of questionnaires was sufficient to represent the SMEs in Saudi Arabia, and they were analyzed using PLS-SEM (Sekaran & Bougie, 2003). A majority of the respondent SME managers were males (68%), with a bachelor’s degree (55.2%), and age 26–30 years (22.4%). The SMEs included in the sample were 75% small and of age groups 1–3 years (21.2%), 4–7 years (23%), and more than 12 years old (29%).

### Measurements

Measures of the constructs of the proposed research model were derived from the literature and modified to suit the nature of this study. To ensure that these measures are valid for these constructs, two of our colleagues reviewed the questionnaire wording. Then, an initial survey was piloted to 15 SME managers in Riyadh. Based on their comments and feedback, some of the questionnaire questions were edited and revised.

The survey constructs were measured using multiple items. All of the questionnaire questions were related to SME business activities and situations since the outbreak of COVID-19 in Saudi Arabia (March 2020). The questionnaire survey was used to examine the impact of SMEs’ innovative responses to the COVID-19 pandemic and external support received during COVID-19 on SMEs’ business performance and survival. The questionnaire consisted of five sections. The first section addressed the personal profile of the enterprise manager and the enterprise characteristics. The second section focused on the innovation practices that have been adopted in the enterprise since the emergence of COVID-19. The third section dealt with enterprise performance after the outbreak of the epidemic. The fourth section addressed project survival indicators, and the fifth section focused on the usage of external support packages provided by the government and NGOs.

#### Dependent variables

Our dependent variables are SME performance and business survival. SME performance was measured using a subjective scale adapted from Bouchikhi (1993), Miller et al. (1988) and O’Farrell (1986). The components of the scale include items related to enterprise sales, profit, assets, capital, production, and market share. These items were measured using a five-point Likert scale (1–largely decreased, 2–decreased, 3–no change, 4–increased, and 5–largely increased).

Business survival was measured using two set indicators (financial indicator and strategy) derived from Barbosa (2016). The financial indicator included five items used to measure an enterprise’s cash availability, debt magnitude, reserved cash, accounts receivable turnover, and technology usage. The strategy indicator comprised five items used to measure the nature of the enterprise’s products, market geographical area, and market segment, ability to estimate sales, and risk tolerance. All of the items were measured using a five-point Likert scale (ranging from 5–strongly agree to 1–strongly disagree).

#### Independent variables

Innovation practices are presented as the main independent variable in the research model. It comprises five sub-constructs adapted from Crossan and Apaydin (2010). Innovation practices usually pertain to new actions and innovation that encourage enterprise internal environmental features (Aragón-Correa et al., 2007). The measures of enterprise innovation practices were made up of several indicators related to enterprise internal settings that operated individually or simultaneously. The five indicators of SME innovation practices embrace “external knowledge,” “structure,” “leadership,” “regeneration,” and “employee’s activities.” All of the items were measured using a five-point Likert-scale (ranging from 5–strongly agree to 1–strongly disagree).

External knowledge is indicated by knowledge and information obtained as a result of existing within social business-related networks in addition to other types of knowledge required to develop enterprise innovation capabilities (Crossan & Apaydin, 2010). External knowledge (six items) was derived from a scale developed by Martensen et al. (2007), Saunila et al. (2014), and Smith et al. (2008).

The structures are related to the required system, work organization, and task arrangement to ensure the success of innovation implementation (Martínez-Román et al., 2011; Smith et al., 2008). The structure construct is divided into sub-constructs in relation to business expenses and production. The six items for expenses and the six items for production sub-constructs were developed from Adams et al. (2006).

The leadership construct was concerned with the support and the encouragement that an enterprise managerial leadership devotes to innovation (Saunila et al., 2014; Smith et al., 2008). The leadership (seven items) scale was modified from Adams et al. (2006).

Regeneration concerns the extent to which the enterprise is able to learn lessons from the past and benefit from previous experiences in developing current innovations (Saunila et al., 2014; Smith et al., 2008). The regeneration (five items) scale was derived from Crossan and Apaydin (2010).

The employees’ activity construct indicates the innovation capabilities of the employees and their enthusiasm and motivation to come up with successful, innovative ideas in different enterprise-related fields (Saunila et al., 2014; Smith et al., 2008). The employees’ activity (five items) scale was derived from Crossan and Apaydin (2010).

#### Moderating variable

External support was inserted into the theoretical model as a moderator in the relationship between innovation practices and SMEs’ performance and innovation practices and business survival. The hypothetical task of external support in this case is to assist the innovative efforts of SMEs, exerted since the COVID-19 pandemic crisis, to reflect positively on its performance and survival. External support was measured through a seven-item scale obtained from official websites of government agencies and NGO websites. These measures concern the types of support that are provided to SMEs during the crisis period. All items were measured using a five-point Likert-scale (ranging from 5–strongly agree to 1–strongly disagree).

## Data analysis and results

### Data analysis

The research hypotheses were tested through the partial least squares structural equation modeling (PLS-SEM) using the SmartPLS 3.2.9 software (Ringle et al., 2005). PLS-SEM is efficient in measuring the strength of structural and complex relationships between model constructs, determining the interaction effect of moderating variables and examining the theoretical soundness of relationships between variables (Chin et al., 2003). Initially, SmartPLS was used to estimate the measurement model-for-model constructs, and then, it was exploited to test hypothetical connections between the latent variables shown in the structural model (Hair Jr et al., 2016).

### Measurement model

The measurement model was tested for reflective and latent variables to ensure the validity of the model’s constructs. Construct validity was evaluated using factor loadings, composite reliability (CR), average variance extracted (AVE), and discriminant validity (Hair Jr & Lukas, 2014). Items of indicators’ loadings, and constructs CR, and AVE are shown in Table 1. Most items exhibited a loading greater than 0.60 (Bagozzi & Yi, 1988) except for item structure (expenses) item STREX5, external support items EXS1 and EXS2, and survival construct item SurvStr1. Eight items (EK1, EK2, EK4, REGEN1, STREX4, STREX5, STREX6, SurFin6) from different constructs with loadings less than 0.50 were deleted to improve construct reliability. Results in the table indicates CR values exceed the criterion (070) as suggested by Hair Jr et al. (2014), ranging between 0.932 and 0.793. Regarding the AVE, results showed that all constructs scored values above the threshold of 0.50 (Hair Jr et al., 2014). The discriminant validity is confirmed since values depicted in Table 2 indicate that the square of the variable correlations with other factors are less than the square root of its AVE (Fornell & Larcker, 1981).

**Table 1:** Internal consistency, convergent validity, composite reliability, and AVE

Construct	Items	Loadings	CR	AVE
External knowledge	EK3	0.700	0.800	0.572
	EK5	0.821		
	EK6	0.743		
Employees’ activities	EMA1	0.806	0.932	0.732
	EMA2	0.896		
	EMA3	0.914		
	EMA4	0.855		
	EMA5	0.802		
Leadership	LEAD1	0.624	0.897	0.557
	LEAD2	0.775		
	LEAD3	0.782		
	LEAD4	0.812		
	LEAD5	0.792		
	LEAD6	0.732		
	LEAD7	0.690		
Expenses	STREX1	0.811	0.805	0.518
	STREX2	0.808		
	STREX3	0.746		
	STREX5	0.453		
Production	STRP1	0.782	0.840	
	STRP2	0.805		
	STRP3	0.728		
	STRP4	0.644		
	STRP5	0.610		
	STRP6	0.782		
Regeneration	REGEN2	0.679	0.800	0.504
	REGEN3	0.560		
	REGEN4	0.817		
	REGEN5	0.758		
External support	EXS1	0.513	0.880	0.527
	EXS2	0.515		
	EXS3	0.616		
	EXS4	0.546		
	EXS5	0.926		
	EXS6	0.879		
Business performance	FINPER1	0.763	0.915	0.645
	FINPER2	0.686		
	FINPER3	0.824		
	FINPER4	0.866		
	FINPER5	0.789		
Financial indicators	SurvFin1	0.704	0.840	0.516
	SurvFin2	0.849		
	SurvFin3	0.571		
	SurvFin4	0.782		
	SurvFin5	0.653		
Strategy	SurvStr1	0.516	0.793	0.571
	SurvStr5	0.846		
	SurvStr6	0.856

**Table 2:** Discriminant validity (Fornell and Larcker (1981), criterion)

	BP	PS	EA	EX	EK	ES	FI	Lead	Pro	Reg.	Stra.	Struc.
**Business performance**	**0.803**											
**Business survival**	0.536	**0.525**										
**Employees’ activities**	0.401	0.318	**0.856**									
**Expenses**	−0.150	−0.088	−0.065	**0.720**								
**External knowledge**	0.105	0.032	0.264	0.161	**0.756**							
**External support**	0.121	0.145	0.127	0.136	0.213	**0.726**						
**Financial indicators**	0.462	0.916	0.182	−0.099	−0.107	0.092	**0.719**					
**Leadership**	0.359	0.415	0.479	−0.060	0.300	0.168	0.228	**0.747**				
**Production**	−0.166	−0.141	−0.057	0.496	0.234	0.220	−0.167	−0.050	**0.718**			
**Regeneration**	0.219	0.235	0.437	−0.016	0.310	0.078	0.121	0.509	0.045	**0.710**		
**Strategy**	0.413	0.712	0.337	−0.064	0.243	0.180	0.394	0.511	−0.033	0.298	**0.756**	
**Structures**	−0.154	−0.113	−0.049	0.792	0.263	0.238	−0.135	−0.049	0.912	0.025	−0.032	0**.563**

Values in the diagonal indicate the square root of latent variable AVE, representing the highest value in each column

### Structural model

Following examining the measurement model,  the next step is to examine the hypothetical relationships between the structural model latent variables using PLS-SEM, structural model’s path coefficients of determination (*R*^2^), and model goodness of fit (GoF) (Memon & Rahman, 2014). Then, the interaction effect was determined as a part of moderation analysis. Prior to the structural model analysis, the collinearity between constructs was reviewed using variance inflation factors (VIF). Table 3 illustrated that all independent variables have VIF value less than benchmark 5 (Hair Jr et al., 2016).

**Table 3:** VIF values for inner model

Construct	Business performance	Business survival	Innovation practices	Structures
Employees’ activities			1.411	
Expenses				1.326
External knowledge			1.260	
External support	1.029	1.042		
Financial indicators		1.187		
Innovation practices	1.029	1.373		
Leadership			1.556	
Production				1.326
Regeneration			1.483	
Strategy		1.533		
Structures		1.102		

Structural model examination results in Table 4 present the exogenous latent variable coefficient of determination (*R*^2^). *R*^2^ indicates the degree to which exogenous latent variables explain the variation in the endogenous variables (Hair Jr et al., 2014). Falk and Miller (1992) suggested that the *R*^2^ value for endogenous variables should not be lower than 0.10 while Chin (1998) classified *R*^2^ values into substantial explanation (value =0.67), moderate explanation (value =0.33), and weak explanation (value=0.19). Accordingly, the endogenous variables business survival and business performance have achieved sufficient variance explained values whereas endogenous variable business performance has scored moderate explanation. Therefore, we can conclude that our proposed structural model has sufficient predictive power.

**Table 4:** Variance explained

Construct	***R*** square	***R*** square adjusted
Business performance	0.222	0.216
Business survival	1.517	1.525
Innovation practices	1.166	1.169

In addition, general goodness-of-fit (GoF) was estimated by calculating the square root of product of inner construct average *R*^2^ and outer construct average AVE (Fornell & Larcker, 1981). Wetzels et al. (2009) suggested that fitness of structural model is considered sufficient if GoF ≥ 0.36. Value of GoF estimated for the research structural model is 0.59, showing that it had a satisfactory fit.

### Hypotheses testing

#### Bootstrapping results

**Fig. 2:**
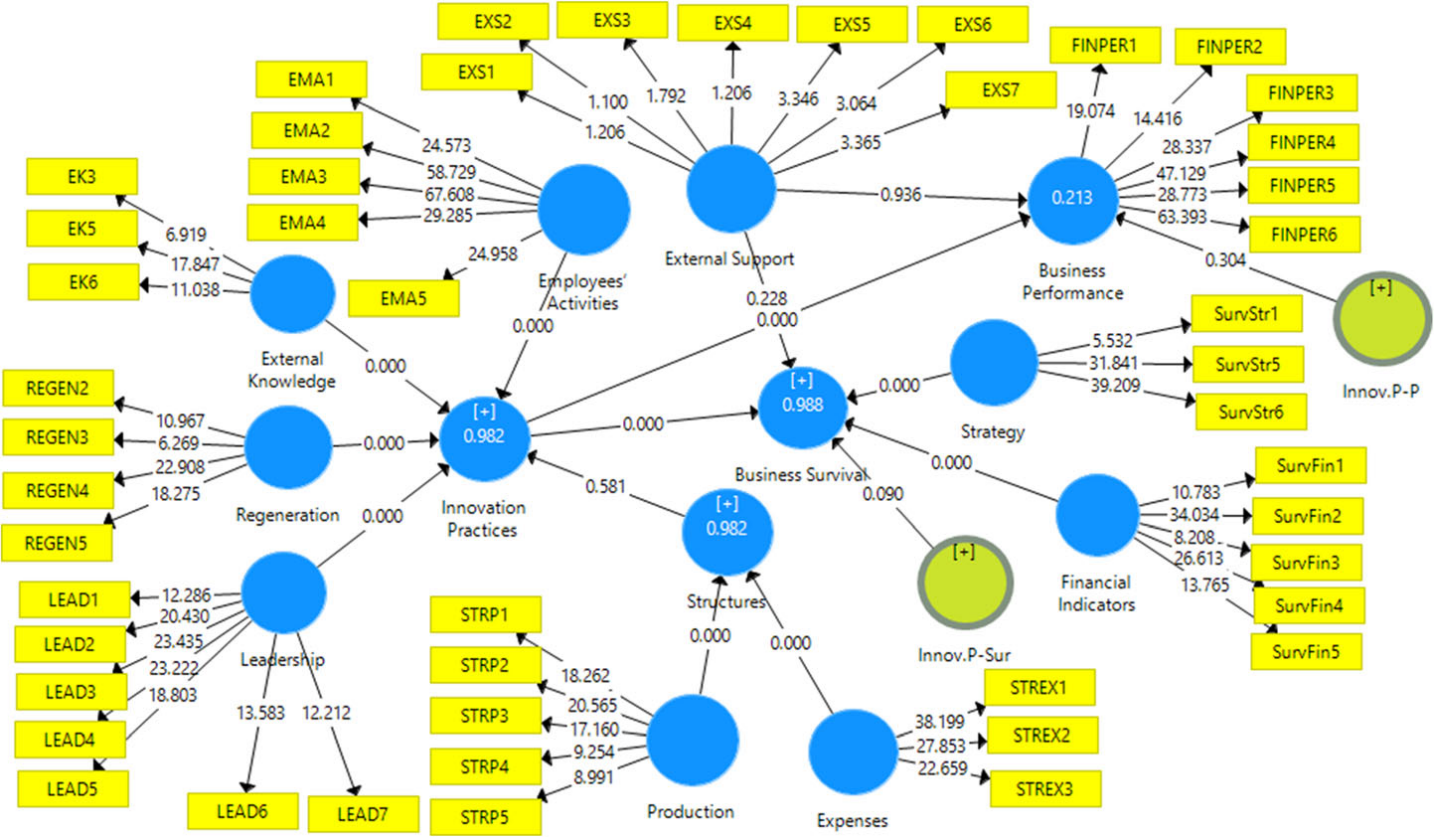
PLS-SEM model with moderating effect

PLS-SEM bootstrapping was used to evaluate the hypothesized relationship among research structural model constructs. The analysis results in Table 5 and Fig. 2 demonstrate path coefficients, significance levels, and *t*-value. Results indicate that hypothesis (H1) is confirmed, and SME innovation practices have significant positive influence on business performance (STD beta= 0.45, *t*=8.432, *p*=0.00). Similarly, hypothesis (H2), which is concerned with the strength of the link between SMEs innovation practices and business survival, is supported (STD beta= 0.054, *t*=3.782, *p*=0.00).

**Table 5:** Hypothesis testing results

Hypothesis	Relationships	Std. beta	Std. error	***t***- value	5% LL	95% UL	Decision
H1	Innovation practices -> business performance	0.45	0.053	8.432**	0.354	0.531	Supported
H2	Innovation practices -> business survival	0.054	0.015	3.782**	0.031	0.078	Supported

**p < 0.01

#### Moderating effect

For analyzing the moderating effects, the variable “external support” was added to the original structural model as a proposed assistant for strengthening the relationship between the independent variable (innovation practices) and the dependent variables (business performance and business survival) (in Fig. 2). PLS-SEM bootstrapping was utilized to examine such moderation relationships. Hypothesis H3 denoted that “external support” has a moderating effect in the relationship between “innovation practices” and “business performance”. Statistical results in Table 6 demonstrates that the moderator variable “external support” has no significant effect in the relationship between “innovation practices” and “business performance”; wherefore, hypothesis H3 is rejected (beta= −0.081, *t*= 1.029, *p*>0.10). Hypothesis H4 is concerned with the strengthening impact of “external support” in the relationship between “innovations practices” and “business survival”. Results exhibited that “external support” has a significant moderation effect in the relationship between “innovation practices” and “business survival”; the *t*-value was found to be 1.698 which is greater than the threshold 1.96, *p*<0.10. Therefore, hypothesis H4 was accepted on a statistical basis.

**Table 6:** Moderating effects

Hypothesis	Relation	STD beta	(STDEV)	***t***-value	***p*** values	5.00%	95.00%	Decision
H3	Innov.P-P -> business performance	−0.081	0.082	1.029	0.304	−0.201	0.084	Not Supported
H4	Innov.P-Sur -> business survival	−0.016	0.013	1.698**	0.09	−0.036	0.008	Supported

**p < 0. 10

## Discussion of results

The SME sector has been significantly affected by the COVID-19 pandemic. During this crisis, SMEs faced difficulties in performing their operational activities and severe financial risks (Omar et al., 2020). Previous studies revealed that SME managers have responded in different ways to the difficulties created by the outbreak of the pandemic (Gerald et al., 2020; Guo et al., 2020; Indriastuti & Fuad, 2020; Sobaih et al., 2021). Additionally, the literature illustrated the importance of external support for performance of SMEs after they have been exposed to the repercussions of the COVID-19 pandemic (e.g., Ahmad et al., 2020; Song et al., 2020). The main purpose of this study is to highlight the importance of “external support” in enhancing the impact of SMEs’ innovation practices as a response to the COVID-19 crisis and its effect on business performance and the likelihood of their survival.

This study was based on a comprehensive model developed to test the moderating role of external support in the relationship between SMEs’ innovation practices adopted during the COVID-19 pandemic and business performance and survival. The results of the study showed that despite the great shock to SMEs caused by the COVID-19 crisis, managers of these enterprises have developed new coping practices. The results of the present study confirmed that the innovation practices of SMEs have a significant and positive impact on business performance (*p*< 0.01). These results indicate that the new management practices (in the field of external knowledge, structures and leadership, regeneration, or employee activities) that have been implemented in SMEs after the COVID-19 outbreak may result in improved performance and increased chances of survival for these enterprises. In other words, SME managers’ intensive communication with others to obtain business information and assistance, including using social media to market their products, spending reductions through workplace sharing and performing tasks online, worker participation in thinking about the business’s future, and active involvement in SME social networks, may positively reflect on the business’s financial performance. These results partially verified the findings of Gerald et al. (2020) on the importance of technology utilization practices to improve SME performance during the COVID-19 pandemic.

The results of the present study also confirmed that the innovative practices that were used by SMEs during COVID-19 significantly affect their likelihood of future survival (*p* < 0.01). Indeed, the research findings indicated that SME survival indicators were positively affected by innovative practices in the fields of external knowledge, structures, leadership, and renewal of employee activities. These findings indicated that SME managers’ intensive communication with others to obtain business information and assistance may increase the likelihood of the business’s survival. The results of the current study supported the findings of Omar et al. (2020), which stated that small business managers have used financial and marketing strategies to ensure that their projects remain relevant in the face of the challenges created by the COVID-19 crisis.

In regard to the study findings, the impact of innovation practices on the performance of SMEs (0.45) outweighs their impact on enterprise survival (0.054). This indicates that managerial innovation practices have a greater impact on an enterprise’s short-term compared to long-term performance. These findings agreed with Freeman (2004), who pointed out that enterprise performance is an outcome of innovation.

The results also indicated that external support provided to SMEs has a significant role in tempering the relationship between innovation practices and enterprise survival. These results indicate that a moderating role of external support is mainly limited to the relationship between innovation practices and enterprise survival, rather than the enterprise short-term performance. The external support provided to SMEs during the COVID-19 pandemic, whether in the form of training, consultancy, or finance, supports the continuity and survival of these enterprises. The findings partially support the arguments of Song et al. (2020) who called on the finance providers to amend their policies to provide SMEs with the required finance to cope with the repercussions of the COVID-19 pandemic crisis. The present study results signify a vital role of external support in strengthening the association between innovation practices undertaken by SMEs during the COVID-19 pandemic crisis and the survival of these enterprises. At the same time, these results denote that this role is less imperative when addressing the relationship between innovation practices and the performance of these enterprises in the short term.

## Implications

This study has implications for SME managers, governments, and policymakers. This study has four implications that may help SME managers mitigate the repercussions of this crisis. First, SME managers should continue to develop creative practices in relation to all enterprise activities to adapt to the challenges imposed by the pandemic. Second, SME managers should keep abreast of necessary business information solutions (whether through networking with other entrepreneurs or consultations and training) to help them make rational decisions to overcome the ordeal. Third, SME managers should constantly update their plans and strategies to achieve the flexibility required to respond to the ramifications of COVID-19. Fourth, because the situation of SMEs after the pandemic will largely differ from their pre-pandemic status, SME managers should develop a strategic business plan to address the negative effects of the crisis on their businesses after the pandemic to ensure continuity and survival.

Additionally, this study provided empirical evidence of the importance of external support (whether governmental or nongovernmental) for the survival of SMEs in times of crisis. As such, this study has important implications for governments and policymakers, who should develop policies to provide more stimulus packages for SMEs that include financing facilities, advisory services, and training. Moreover, governments should encourage NGOs to provide different kinds of support to SMEs in the form of consultations, training, advice, guidance, and psychological support to help them cope with the difficulties caused by COVID-19. Additionally, because the COVID-19 crisis has greatly affected SMEs’ financial position, governments must encourage finance providers to adopt more flexible policies when financing SMEs, such as low-interest loans and the consideration of the enterprise’s financial position for loan installments.

This research also has significant theoretical implications because it developed a comprehensive model to examine the role of external support received by SMEs during the COVID-19 pandemic in moderating the relationship between innovation practices adopted by SMEs and their performance and survival. Thus, this study added to the literature by arguing that in times of crisis similar to COVID-19, external support can help an enterprise obtain more positive results from innovation practices in the form of performance improvements and strengthened survival indicators. Crises usually weaken the performance of SMEs and their ability to survive (Michael & Robbins, 1998; Robbins & Pearce II, 1993), but in this case, external support can push innovation efforts. Likewise, crises usually affect an enterprise’s sales, production capabilities, and financial position. Therefore, the present study proposes that SMEs develop new practices and ideas to obtain knowledge and information from external parties, build effective structures for production and expenditures, follow motivational leadership, and implement effective employee activities to ensure good business performance and protect the future of the enterprise.

## Conclusions

The current research proposes a theoretical model for studying the moderating effect of external support, provided during the COVID-19 epidemic crisis, in strengthening the link between innovation practices and the performance and survival of SMEs using the PLS-SEM algorithm. The study based on four basic hypotheses in relation to the association between these variables. The main findings of the study suggest that the innovation practices of SMEs have a significant impact on the performance and survival of SMEs. Additionally, the study results confirmed the significant and moderating role of external support provided to SMEs during the COVID-19 epidemic crisis and the survival of the business. Results of the study showed that the policies adopted by Saudi’s government to reduce the repercussions of the COVID-19 epidemic crisis on SMEs, which represented numerous financial support packages and encouraged the support of nongovernmental organizations, was expected to contribute to the resilience of these enterprises in facing such a crisis.

Although the current study has achieved findings that have significant implications for SME managers and policy makers, it has some limitations. Because of the wide range of innovation practices, the study focused only on administrative innovation practices and excluded other fields, such as technological innovations. Another limitation of this study is the measurement of the performance of SMEs using financial and marketing indicators and ignoring other indicators, such as administrative, social, and psychological elements.

Future research could expand upon these conclusions by addressing the shortcomings of the current study. Because of the diversity of the sectors to which small enterprises belong, it would be beneficial to conduct a sector-based examination of their practices. Furthermore, to obtain comprehensive and in-depth insight into the nature of the relationship between SMEs’ innovation practices, external support, and business performance and survival, all indicators for measuring enterprise performance should be considered, and the types of innovation must be addressed.

## Data Availability

The datasets generated during and/or analyzed during the current study are included in the article, and the raw data are available from the corresponding author on reasonable request.

## Abbreviations

SMEs: Survival of small and medium enterprises
COVID-19: Coronavirus
PLS-SEM: Partial least squares structural equation modeling
NGOs: Nongovernmental organizations
OECD: Organization for Economic Co-operation and Development
GaStat: General Authority for Statistics database
CR: Composite reliability
AVE: Average variance extracted
GoF: Model goodness of fit
VIF: Variance inflation factors
